# Recent and Recurrent Autopolyploidization Fueled Diversification of Snow Carp on the Tibetan Plateau

**DOI:** 10.1093/molbev/msae221

**Published:** 2024-10-22

**Authors:** Xinxin Li, Min Wang, Ming Zou, Xiaotong Guan, Shaohua Xu, Weitao Chen, Chongnv Wang, Yiyu Chen, Shunping He, Baocheng Guo

**Affiliations:** Key Laboratory of Zoological Systematics and Evolution, Institute of Zoology, Chinese Academy of Sciences, 100101 Beijing, China; Key Laboratory of Zoological Systematics and Evolution, Institute of Zoology, Chinese Academy of Sciences, 100101 Beijing, China; University of Chinese Academy of Sciences, 100049 Beijing, China; Key Laboratory of Zoological Systematics and Evolution, Institute of Zoology, Chinese Academy of Sciences, 100101 Beijing, China; Key Laboratory of Zoological Systematics and Evolution, Institute of Zoology, Chinese Academy of Sciences, 100101 Beijing, China; University of Chinese Academy of Sciences, 100049 Beijing, China; Key Laboratory of Zoological Systematics and Evolution, Institute of Zoology, Chinese Academy of Sciences, 100101 Beijing, China; University of Chinese Academy of Sciences, 100049 Beijing, China; Pearl River Fisheries Research Institute, Chinese Academy of Fishery Science, 510000 Guangzhou, China; Key Laboratory of Zoological Systematics and Evolution, Institute of Zoology, Chinese Academy of Sciences, 100101 Beijing, China; State Key Laboratory of Freshwater Ecology and Biotechnology, Institute of Hydrobiology, Chinese Academy of Sciences, 430072 Wuhan, China; National Natural Science Foundation of China, Beijing 100085, China; University of Chinese Academy of Sciences, 100049 Beijing, China; State Key Laboratory of Freshwater Ecology and Biotechnology, Institute of Hydrobiology, Chinese Academy of Sciences, 430072 Wuhan, China; Key Laboratory of Zoological Systematics and Evolution, Institute of Zoology, Chinese Academy of Sciences, 100101 Beijing, China; University of Chinese Academy of Sciences, 100049 Beijing, China; Academy of Plateau Science and Sustainability, Qinghai Normal University, 810008 Xining, China

**Keywords:** whole-genome duplication, speciation, ohnolog, gene tree, isoform sequencing

## Abstract

Whole-genome duplication (WGD), or polyploidization, is a major contributor to biodiversity. However, the establishment and survival of WGDs are often considered to be stochastic, since elucidating the processes of WGD establishment remains challenging. In the current study, we explored the processes leading to polyploidy establishment in snow carp (Cyprinidae: Schizothoracinae), a predominant component of the ichthyofauna of the Tibetan Plateau and its surrounding areas. Using large-scale genomic data from isoform sequencing, we analyzed ohnolog genealogies and divergence in hundreds to thousands of gene families across major snow carp lineages. Our findings demonstrated that independent autopolyploidization subsequent to speciation was prevalent, while autopolyploidization followed by speciation also occurred in the diversification of snow carp. This was further supported by matrilineal divergence and drainage evolution evidence. Contrary to the long-standing hypothesis that ancient polyploidization preceded the diversification of snow carp, we determined that polyploidy in extant snow carp was established by recurrent autopolyploidization events during the Pleistocene. These findings indicate that the diversification of extant snow carp resembles a coordinated duet: first, the uplift of the Tibetan Plateau orchestrated the biogeography and diversification of their diploid progenitors; then, the extensive Pliocene–Pleistocene climate changes acted as relay runners, further fueling diversification through recurrent autopolyploidization. Overall, this study not only reveals a hitherto unrecognized recent WGD lineage in vertebrates but also advances current understanding of WGD processes, emphasizing that WGD establishment is a nonstochastic event, emerging from numerous adaptations to environmental challenges and recurring throughout evolutionary history rather than merely in plants.

## Introduction

Polyploidization, or whole-genome duplication (WGD), is widely regarded as a key driver of genetic innovation, biological complexity, and species diversification (Van de Peer et al. 2009, 2017, 2021; Fox et al. 2020). The timing of WGD events often aligns with significant climatic and geological changes, such as the Cretaceous–Paleogene extinction event (Vanneste et al. 2014; Koenen et al. 2021; Wu et al. 2020), recent glaciation maxima (Estep et al. 2014; Novikova et al. 2018), and temperature extremes (Rice et al. 2019; Novikova et al. 2020), suggesting that WGDs play an adaptive role in evolution (Van de Peer et al. 2009, 2017, 2021; Fox et al. 2020). However, the immediate detrimental effects of WGDs on fertility and fitness, such as mitotic and meiotic abnormalities and genomic instability (Ramsey and Schemske 2002; Comai 2005; Madlung et al. 2005; Prieto and Naranjo 2020), as well as the rarity of WGD lineages across the tree of life (Arrigo and Barker 2012; Vanneste et al. 2014; Mayrose et al. 2015), have led to the suggestion that their establishment and persistence may be evolutionary coincidences (Meyers and Levin 2006). This is particularly evident in vertebrates, where, despite two known WGD events in the ancestral vertebrate lineage potentially driving vertebrate evolution (Ohno 1970; Dehal and Boore 2005), WGDs remain rare in modern vertebrates, with the exceptions of amphibians and fish (David 2022). The challenge in determining whether the establishment and long-term survival of WGDs in nature are adaptative or random arises largely from the fact that most known WGD lineages are ancient, dating earlier than 50 million years ago (Mya), with recurrent WGDs rarely identified in these lineages (Van de Peer et al. 2017). In contrast, recurrent WGDs appear to be more prevalent and nonrandom in younger evolutionary lineages, such as in sorghum (Andropogoneae) occurring around 11.60 to 5.30 Mya (Estep et al. 2014), cabbage (Brassicaceae) occurring around 12.00 to 7.00 Mya (Kagale et al. 2014), and sunflower (Asteraceae) occurring around 23.00 to 11.60 Mya (Huang et al. 2016). Therefore, the ongoing debate about whether WGDs in nature are random events or adaptive responses primarily stems from our limited understanding of the evolutionary processes involved in the establishment of WGD lineages.

**Fig. 1.**
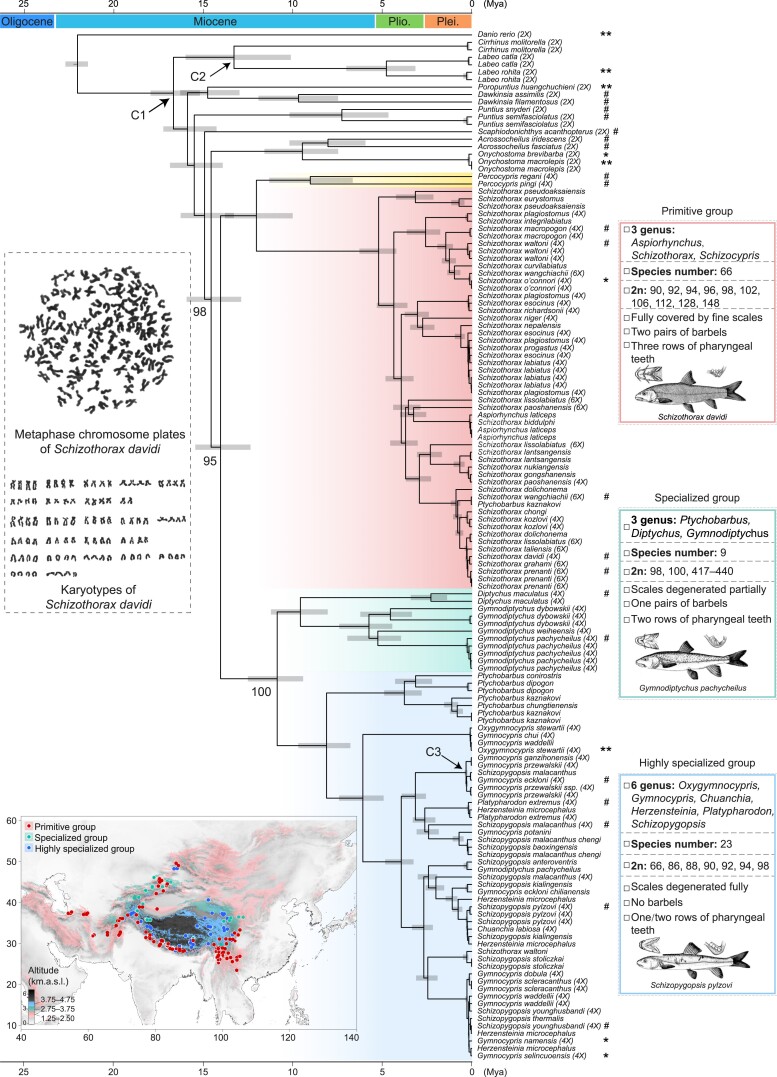
Morphological characters, geographical occurrence, and matrilineal phylogeny and divergence of snow carp. Genera, species number, karyotype, and morphological characters of snow carp in primitive, specialized, and highly specialized groups. Body and head diagrams and pharyngeal teeth diagrams were adapted from (Chen 1998; Chang and Miao 2016), respectively. Geographical occurrence records of snow carp. Elevation data (spatial resolution 30 arcseconds) were retrieved from WorldClim (Fick and Hijmans 2017). Time-calibrated mitochondrial phylogeny estimated using MCMCTree with approximate likelihood for 78 Cyprinidae species. The bars on the nodes represent 95% highest posterior density credibility interval of node age. Numbers are bootstrap values. C1, minimum age of 16.00 Mya for MRCA of Barbinae and Labeoninae; C2, minimum age of 5.33 Mya for root of Labeoninae; C3, maximum age of 0.15 Mya for MRCA of *Gymnocypris przewalskii* and *Gymnocypris eckloni*. Known ploidy is given in parentheses. Species with de novo Iso-seq data which are determined in this study are labelled with #, and species with de novo Iso-seq data and genome from public dataset are marked as * and **, respectively. Metaphase of cell division and karyotype (2*n* = 98) of *Schizothorax davidi* are shown.

The origin and evolution of biodiversity associated with the Tibetan Plateau are still not well understood, despite an increasing number of studies (Favre et al. 2015). Climatic oscillations since the late Pliocene are believed to promote polyploidy speciation including both allopolyploids and autopolyploids in plants on the Tibetan Plateau (Wang et al. 2017). In fact, polyploids are commonly found not only in plants but fishes (David 2022) on the Tibetan Plateau. Snow carp (Cyprinidae: Schizothoracinae) comprise more than 100 currently recognized species from 12 genera (Wu and Wu 1992; Chen and Cao 2000) and are the major component of ichthyofauna on the Tibetan Plateau and surrounding areas. Traditionally regarded as a monophyletic group, snow carp are thought to have originated from primitive Barbinae on the Tibetan Plateau during the late Tertiary (23.03 to 2.58 Mya), with expansion to their current habitats following the uplift of the Tibetan Plateau (Das and Subla 1963; Cao et al. 1981; Chen and Cao 2000). Based on their morphological characteristics (Fig. 1) and altitudinal distributions (Fig. 1), snow carp are categorized into primitive, specialized, and highly specialized groups (Cao et al. 1981). The diversification of snow carp is consistent with the uplift phases of the Tibetan Plateau, as evidenced by molecular phylogenetic studies (He et al. 2004; He and Chen 2006, 2007; Gaubert et al. 2009; Zhao et al. 2009). Furthermore, the temporal distribution patterns of their fossil counterparts closely mirror their current spatial distribution, exemplifying local endemism origination on the Tibetan Plateau and reflecting the biological responses to its stepwise uplift (Favre et al. 2015; Deng et al. 2020). Remarkably, all known snow carp are polyploids, exhibiting a range of ploidy levels from tetraploid to some species having up to 470 chromosomes (Zan et al. 1985; Zhang, Tang et al. 2016). This suggests a much more complex evolutionary history for extant snow carp than the previously held view of a single, Barbinae-like tetraploid species as their common ancestor (Yang et al. 2015, 2022). Snow carp thus provide a unique opportunity to explore the establishment and diversification of WGD lineages in vertebrates. While large-scale genomic data have been applied to a few species (Li and Guo 2020; Xiao et al. 2020; Tian et al. 2022), our current understanding of snow carp evolution predominantly relies on mitochondrial DNA sequences (Yonezawa et al. 2014; Yang et al. 2015, 2022; Zhang, Chen et al. 2016; Bibi et al. 2019). Nevertheless, molecular phylogenetics indicates that snow carp are nonmonophyletic (Yonezawa et al. 2014; Yang et al. 2015, 2022; Tan and Armbruster 2018), contrary to conclusions drawn from morphological studies (Cao et al. 1981). Consequently, comprehending the establishment and diversification of this polyploid vertebrate lineage amidst the palaeogeological and paleoclimatic fluctuations accompanying the uplift of the Tibetan Plateau remains a largely unexplored area, both from macroecological and macroevolutionary perspectives.

In the current study, we explored the evolutionary establishment process of snow carp by leveraging large-scale genomic data. We first investigated whether extant snow carp originated from independent and/or shared polyploidization events by inferring ohnolog genealogies in hundreds to thousands of gene families among snow carp species. Subsequently, we examined whether extant snow carp resulted from autopolyploidization and/or allopolyploidization by reconciling topologies of thousands of gene trees and estimating ohnolog divergence. Our findings indicate that snow carp are among the youngest known polyploid lineages, with their establishment and diversification primarily driven by recent and recurrent autopolyploidization events. Finally, we proposed a scenario for the progressive establishment of polyploid snow carp from both macroecological and macroevolutionary perspectives by integrating their matrilineal divergence, polyploidization events, and fossil occurrences with paleoclimate fluctuations, suggesting that polyploid snow carp repeatedly emerged and ultimately outcompeted their diploid progenitors during the extensive Pliocene–Pleistocene climate changes. Collectively, our study reveals a novel evolutionary paradigm, supporting the hypothesis that the establishment of polyploids is an adaptive response not only in plants but also in vertebrates, rather than a random occurrence, in the face of global geological and climatic changes.

## Results

### Mitochondrial Perspective on Snow Carp Diversification

Based on analysis of mitochondrial genomes, we identified two divergent clades in the extant snow carp (Fig. 1; supplementary table S1, Supplementary Material online), one comprising *Percocypris* and *Schizothorax* from the primitive group, and the other including all members of the specialized and highly specialized groups, consistent with earlier studies (Yang et al. 2015, 2022; Li and Guo 2020). The estimated matrilineal origin of snow carp was approximately 14.57 Mya (95% confidence interval [CI] 15.94 to 12.89), with a divergence of the two snow carp clades occurring approximately 14.04 Mya (95% CI 15.45 to 12.36). *Schizothorax* diverged from *Percocypris* around 12.03 Mya (95% CI 13.79 to 10.01), with its diversification beginning at about 5.20 Mya (95% CI 6.27 to 4.21). The specialized and highly specialized groups diverged from each other approximately 10.87 Mya (95% CI 12.50 to 9.42), with diversification within these two groups commencing around 9.57 Mya (95% CI 11.22 to 8.05) and 8.11 Mya (95% CI 9.69 to 6.78), respectively. The matrilineal origin and diversification timeline of the extant snow carp coincided with the continuous uplift of the Tibetan Plateau and concurrent climatic changes since the Middle Miocene (Favre et al. 2015; Wang et al. 2016; Deng et al. 2020; Li and Guo 2020).

### Multiple Independent and Recent Polyploidization Events in Snow Carp Diversification

**Fig. 2.**
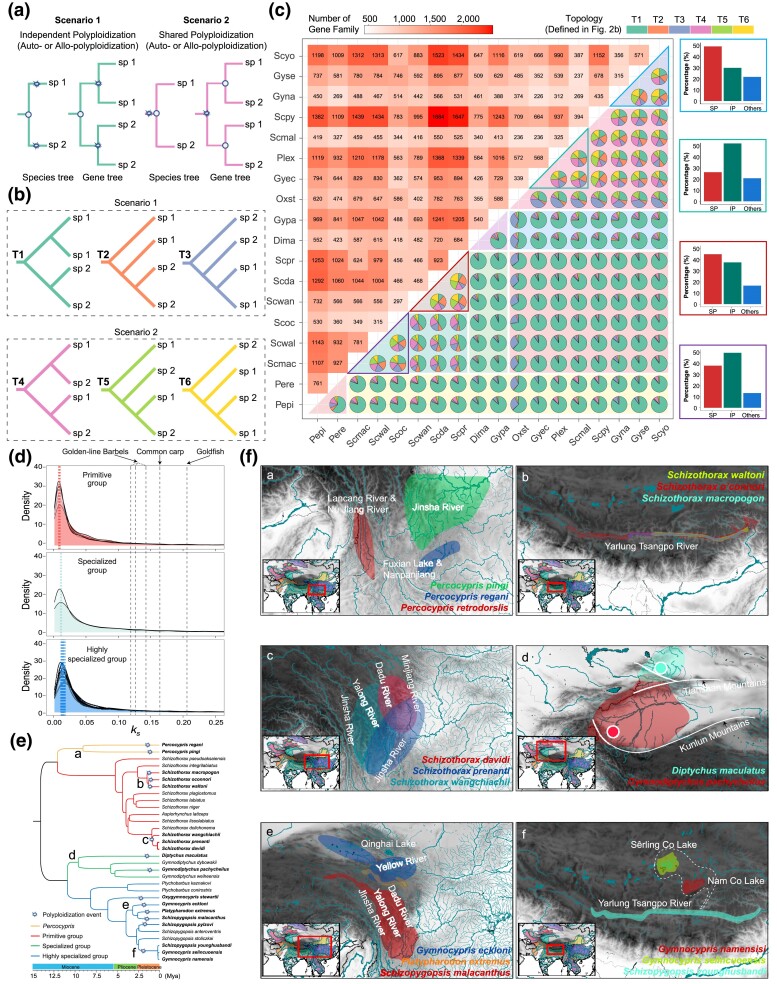
Phylogenomic and biogeographical evidence for multiple independent polyploidization events in snow carp diversification. a) Two theoretical scenarios of ohnologous genealogies in two closely related tetraploids. Stars represent polyploidization events and circles represent speciation events. b) Six observed topologies of ohnologous genealogies in two closely related tetraploids, focusing on species rather than ohnologous identity. c) Ohnologous genealogies within each snow carp. Numbers in upper left panel are orthologous gene families used in ohnologous genealogy inference for each pair of snow carp. Pie charts in lower right panel are frequencies of six observed topologies for ohnologous genealogies for each pair of snow carp. Each bar plot shows frequencies of ohnologous genealogies supporting independent polyploidization (IP) and shared polyploidization (SP) in snow carp triplet. Abbreviations for each species are given in supplementary table S1, Supplementary Material online. d) Density distribution of *K*_S_ values between ohnologs in snow carp. Dashed lines demonstrate peak of *K*_S_ density distribution in ohnologs in allopolyploids of golden-line cavefish (Li and Guo 2020), common carp (Xu et al. 2014; Xu et al. 2019), and goldfish (Chen et al. 2019; Luo et al. 2020), respectively. e) Polyploidization events identified in snow carp in this study. Topology and divergence time are adopted from Fig. 1. f) Current geographical distribution of snow carp in nodes a-f in Fig. 2e. Major river basins on the Tibetan Plateau and surrounding areas were retrieved from the World Bank (https://datacatalog.worldbank.org/search/dataset/0041426).

The polyploidization history of snow carp was investigated to clarify their evolutionary divergence. Specifically, the relationship of ohnologs (duplicated genes from polyploidization) within and between species was examined to infer the timing of polyploidization events in relation to speciation. In two closely related tetraploid species, the arrangement of ohnologs can indicate the sequence of polyploidization and speciation events. Of note, if independent polyploidization occurred after speciation (scenario #1, Fig. 2a), ohnologs within each species would group together. In contrast, if speciation occurred after polyploidization (scenario #2, Fig. 2a), ohnologs would group across the two species. Here, we identified 15 possible topologies among ohnologs in an orthologous gene family in two tetraploid species (supplementary fig. S1, Supplementary Material online). These were simplified into six main topologies (T1 to T6) by focusing on species instead of individual ohnolog identities (Fig. 2b). Topologies T1 to T3 suggested that two tetraploids originated from independent polyploidization events, while T4 to T6 indicated a shared polyploidization event (Fig. 2b). To ascertain whether snow carp arose from independent or shared polyploidization events (Fig. 2a), we analyzed ohnologous genealogy in each orthologous gene family across pairs from a total of 18 snow carp species, covering 226 to 1,684 orthologous gene families (Fig. 2c; supplementary tables S2 and S3, Supplementary Material online). The dominance of topologies T1 to T3 in 141 out of 153 pairwise inferences (Fig. 2c; supplementary table S4, Supplementary Material online) suggested that the diversification of snow carp was predominantly driven by independent polyploidization events (scenario #1, Fig. 2a).

Our results indicated that snow carp from the same genus underwent independent polyploidization events, as described in scenario #1 (Fig. 2a). Specifically, a significant proportion (78.71%, 599/761) of ohnologous genealogies (Fig. 2c; supplementary table S4, Supplementary Material online) demonstrated independent polyploidization in the two *Percocypris* species (node a, Fig. 2e). The synonymous substitution rate (*K*_s_) distribution peaks between ohnologs were 0.0065 in *Percocypris pingi* and 0.0102 in *Percocypris regani* (top panel, Fig. 2e; supplementary table S5, Supplementary Material online), corresponding to divergence times of 0.93 Mya and 1.45 Mya, respectively, suggesting recent and independent polyploidization events in these two species rather than a shared ancient autopolyploidization event (Gu et al. 2024). Additionally, the matrilineal divergence between the two *Percocypris* species (9.02 Mya, 95% CI 11.31 to 6.65, supplementary table S5, Supplementary Material online; node a, Fig. 2e) coincided with drainage divergence on the southeastern Tibetan Plateau (Cui and Chu 1990), the native region of these species (panel a, Fig. 2f). These findings imply that polyploidization occurred independently in the two *Percocypris* species, long after matrilineal and biogeographical divergence from their diploid progenitors. Independent polyploidization, as outlined in scenario #1 (Fig. 2a), was also evident in the genus *Schizothorax* (node b, Fig. 2e). This observation held true whether ohnolog genealogies were inferred between each pair of the three *Schizothorax* species (pie charts within the purple triangle box, Fig. 2c) or collectively among all three species (bar plot in the purple quadrilateral box, Fig. 2c). Based on ohnolog divergence within each of the three *Schizothorax* species (top panel, Fig. 2d; 1.14 to 1.05 Mya, supplementary table S5, Supplementary Material online), polyploidization occurred shortly after their matrilineal divergence (1.44 Mya with 95% CI 1.92 to 1.05 and 1.25 Mya with 95% CI 1.67 to 0.88, supplementary table S5, Supplementary Material online; node b, Fig. 2e). These polyploidization events likely occurred in geographical isolation of snow carps within distinct paleo-lakes, coinciding temporally with the C phase of the Qing-Zang tectonic movement (1.80 to 1.40 Mya) during the stepwise uplift of the Tibetan Plateau (Li and Fang 1999). Later on, the connection between those isolated paleo-lakes and the form Yalung Tsangpo River (Liu and Sun 2007; Li et al. 2015) may have facilitated the current sympatric distribution of the three *Schizothorax* species in the Yarlung Tsangpo River (panel b, Fig. 2f).

Our results also showed that snow carp from different genera within a matrilineally monophyletic group underwent independent polyploidization events, aligning with scenario #1 (Fig. 2a). Notably, more than 90.56% of ohnologous genealogies (489/540 orthologous gene families, Fig. 2c; supplementary table S4, Supplementary Material online) demonstrated the occurrence of independent polyploidization in *Diptychus maculatus* and *Gymnodiptychus pachycheilus* from the specialized group (node d, Fig. 2e). The *K*_s_ distribution peaks between ohnologs were 0.0094 in *Diptychus maculatus* and 0.0078 in *Gymnodiptychus pachycheilus* (middle panel, Fig. 2d; supplementary table S5, Supplementary Material online), corresponding to divergence times of 1.34 Mya and 1.11 Mya, respectively, suggesting that polyploidization in these two species occurred recently and nearly simultaneously. The matrilineal divergence between *Diptychus maculatus* and *Gymnodiptychus pachycheilus* (9.57 Mya, 95% CI 11.22 to 8.05, supplementary table S5, Supplementary Material online; node d, Fig. 2e) coincided with the geological separation (10.00 to 8.00 Mya) of the north Tianshan Mountain River system and Kunlun Mountains (Wang et al. 2003), where these two species are respectively distributed (panel d, Fig. 2f), indicating that polyploidization in these species occurred independently, well after matrilineal and biogeographical divergence from their diploid progenitors. Similarly, independent polyploidization was also observed in *Gymnocypris eckloni*, *Platypharodon extremus*, and *Schizopygopsis malacanthus* from the highly specialized group (node e, Fig. 2e; supplementary table S4, Supplementary Material online). This was consistent whether ohnolog genealogies were inferred between each species pair (pie charts in green triangle box, Fig. 2c) or among the three species collectively (bar plot in green quadrilateral box, Fig. 2c). Ohnolog divergence within each species (bottom panel, Fig. 2d; 1.82 to 1.44 Mya, supplementary table S5, Supplementary Material online) also suggested that polyploidization occurred independently in the three species after their matrilineal divergence (3.14 Mya, 95% CI 3.91 to 2.56, supplementary table S5, Supplementary Material online; node e, Fig. 2e). Stratigraphic evidence indicates that the main drainages of the upper Yellow River and the Yangtze River, where these three species are distributed (panel e, Fig. 2f), existed as a series of isolated ancient lake basins until 1.90 to 1.70 Mya (Li et al. 2015). Therefore, polyploidization in these three species likely occurred independently in different drainages where their diploid progenitors were distributed and isolated.

Our analyses further revealed that polyploidization preceding speciation, as per scenario #2 (Fig. 2a), occurred recurrently during snow carp diversification. In the genus *Schizothorax*, evidence of shared polyploidization was found among *Schizothorax wangchiachii*, *Schizothorax prenanti*, and *Schizothorax davidi* (node c, Fig. 2e). This was supported regardless of whether ohnolog genealogies were inferred between each species pair (pie charts in red triangle box, Fig. 2c; supplementary table S4, Supplementary Material online) or considered collectively among the three species (bar plot in red quadrilateral box, Fig. 2c). Furthermore, ohnolog divergence within each of the three *Schizothorax* species (top panel, Fig. 2d; 0.93 to 0.88 Mya, supplementary table S5, Supplementary Material online) indicated that polyploidization preceded matrilineal divergence (0.78 Mya with 95% CI 1.04 to 0.56 and 0.22 Mya with 95% CI 0.28 to 0.16, supplementary table S5, Supplementary Material online; node c, Fig. 2e), suggesting shared polyploidization among these three species. At present, the three *Schizothorax* species are sympatrically distributed in the upper Yangtze River drainages (panel c, Fig. 2f). Similarly, shared polyploidization events were inferred among *Gymnocypris namensis*, *Gymnocypris selincuoensis*, and *Schizopygopsis younghusbandi* (node f, Fig. 2e), as indicated by ohnolog genealogy analyses between each species pair (pie charts in blue triangle box, Fig. 2c; supplementary table S4, Supplementary Material online) and among all three species collectively (bar plot in blue quadrilateral box, Fig. 2c). The shared polyploidization between the genera *Gymnocypris* and *Schizopygopsis* is understandable, given the existence of paraphyly between them (including the species *Schizopygopsis younghusbandi*) (Tang et al. 2019). Ohnolog divergence within each of the three species (bottom panel, Fig. 2d; 2.32 to 1.58 Mya, supplementary table S5, Supplementary Material online) demonstrated that polyploidization occurred well before their matrilineal divergence (0.16 Mya, 95% CI 0.21 to 0.11, supplementary table S5, Supplementary Material online; node f, Fig. 2e), coinciding with the last connection of Nam Co Lake, Sêrling Co Lake, and the Yarlung Zangbo River, a period marked by the existence of extensive pan-lakes between 0.20 and 0.04 Mya (Zheng et al. 2005). These results indicate that the present distribution of the three species in isolated drainages (panel f, Fig. 2f) resulted from allopatric speciation of a common polyploid progenitor.

We identified a total of 14 independent polyploidization events in the 18 snow carp species studied (Fig. 2e). Independent polyploidization was strongly supported by both ohnolog genealogy between snow carp species (Fig. 2c) and ohnolog divergence within snow carp species, as evidenced by the distinct *K*_S_ density distribution peaks for intraspecific ohnolog divergence (Fig. 2d; supplementary table S5, Supplementary Material online). The timing of matrilineal divergence among snow carp species, which coincided with disconnection between their current drainages, was significantly earlier than the ohnolog divergence in each species in scenarios of independent polyploidization following speciation (nodes a, b, d, and e in Fig. 2e; panels a, b, d, and e in Fig. 2f). Conversely, in cases of shared polyploidization occurring before speciation (nodes c and f, Fig. 2e; panels c and f, Fig. 2f), the matrilineal divergence occurred later than ohnolog divergence, providing compelling evidence for independent polyploidization events in the evolution of snow carp. The range of density distribution peaks for intraspecific ohnolog divergence spanned from 0.0064 to 0.0141 (Fig. 2d; supplementary table S5, Supplementary Material online). This finding aligns with previous genome-wide studies (Wen et al. 2020; Xiao et al. 2020) but is markedly smaller than the divergence observed in common carp (0.1650) (Xu et al. 2019; Wen et al. 2020), goldfish (0.2050) (Chen et al. 2019; Luo et al. 2020), and golden-line cavefish (0.1210 to 0.1420) (Li and Guo 2020). This indicates that the polyploidization events in snow carp are relatively recent compared with other well-known polyploid Cyprinidae lineages. Overall, our study illustrates that diversification of snow carp involved multiple, recent independent polyploidization events.

### Recurrent Autopolyploidization in Snow Carp Diversification

**Fig. 3.**
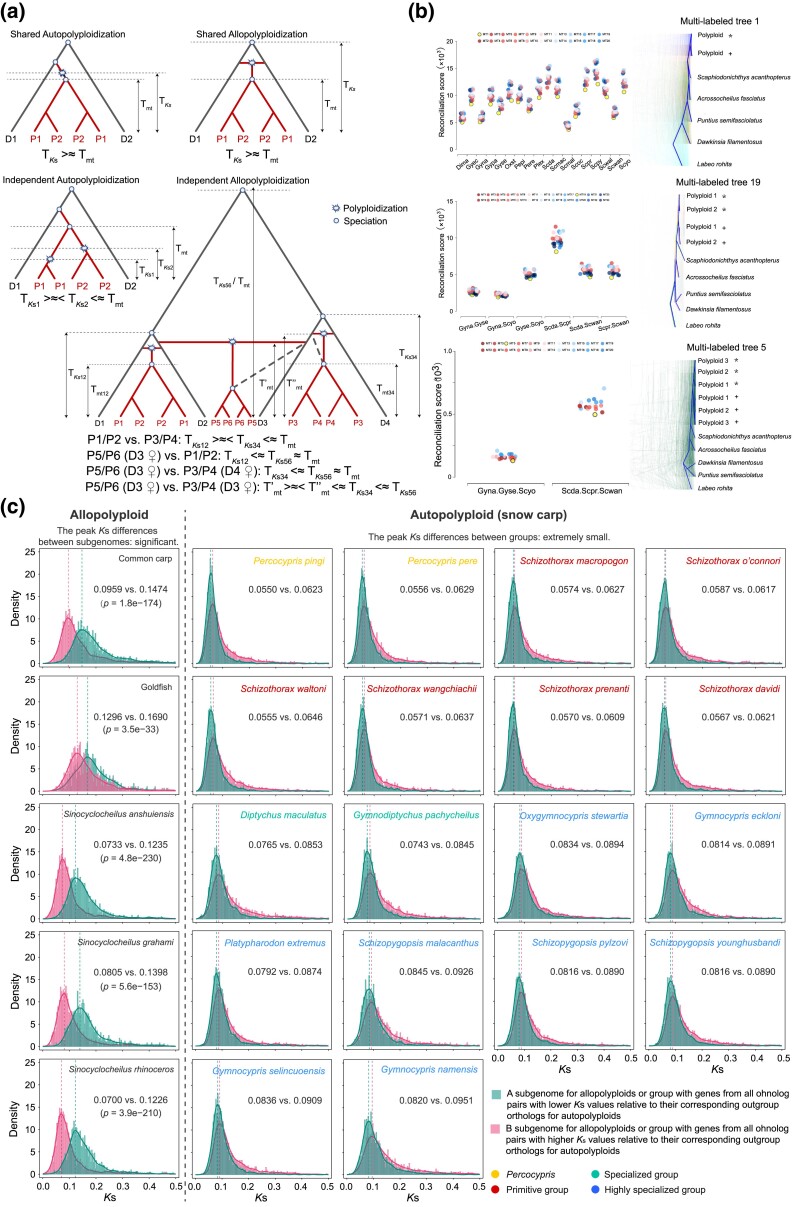
Comparisons between ohnologs in snow carp and their orthologs in diploid Cyprinidae species, demonstrating recurrent autopolyploidization in snow carp diversification. a) Relationship between intraspecific ohnologous divergence and interspecific mitochondrial divergence in closely related auto- and allopolyploids. T*_K_*_s_, divergence time between ohnologs within each polyploid species; T_mt_, mitochondrial divergence time between polyploid species. D1 to D4, diploid species; P1 to P6, polyploid species. b) GRAMPA results. Reconciliation scores (scatter plots) of MUL-trees (MT) with one snow carp (top panel), two snow carp from a shared polyploidization event (middle panel), and three snow carp from a shared polyploidization event (bottom panel), and topologies of MUL-trees with lowest reconciliation scores, respectively. Abbreviations for each species are given in supplementary table S1, Supplementary Material online. c) Density distribution of *K*_S_ values between ohnologs in polyploid Cyprinidae species and their orthologs in diploid Cyprinidae species. *K*_S_ density distribution in two subgenomes (a and b) is shown in common carp, goldfish, and golden-line cavefish, respectively. For each snow carp, *K*_S_ density distribution is shown for two arbitrary groups, where “L” includes all larger *K*_S_ values from each pair of orthologs and “L” includes all those with smaller values.

Comparisons between intraspecific ohnologous divergence and interspecific mitochondrial divergence (Fig. 3a) in closely related snow carp (Figs. 1c and 2c; supplementary table S5, Supplementary Material online) demonstrated that either independent auto- or allopolyploidization likely occurred in nodes a, b, d, or e (Fig. 2e) and either shared auto- or allopolyploidization likely occurred in nodes c or f (Fig. 2e). To ascertain whether auto- and/or allopolyploidization contributed to the diversification of snow carp, ohnologous gene trees—multilabeled (MUL) trees were reconciled with singly labeled species trees (supplementary fig. S2, Supplementary Material online), involving their closest diploid relatives (Yang et al. 2015, 2022), using GRAMPA—the Gene-tree Reconciliation Algorithm with MUL-trees for Polyploid Analysis (Thomas et al. 2017). Reconciliation of the ohnologous gene trees to singly labeled species tree was first conducted for each of the 18 snow carp species, resulting in the reconciliation of 760 to 2,526 ohnologous gene trees (supplementary fig. S3, Supplementary Material online). For each species, the autopolyploid MUL-tree (top-right panel, Fig. 3b) consistently showed the lowest reconciliation score (top-left panel, Fig. 3b), suggesting autopolyploid origins for the snow carp. Subsequently, reconciliation of ohnologous gene trees to singly labeled species tree was conducted for snow carp associated with shared polyploidization events (nodes c and f, Fig. 2e). Similarly, autopolyploid MUL-trees (middle-right and bottom-right panels, Fig. 3b) consistently yielded the lowest reconciliation scores (middle-left and bottom-left panels, Fig. 3b). This analysis, encompassing the reconciliation of 380 to 1,661 ohnologous gene trees (supplementary fig. S3, Supplementary Material online) for each of the six pairs and two triplets of snow carp, suggested that both events (nodes c and f, Fig. 2e) were instances of autopolyploidization. Therefore, the comprehensive reconciliation of ohnologous gene trees with singly labeled species trees across all studied snow carp points to a predominantly autopolyploid origin for these species.

Despite incorporating the closest known diploid relatives of snow carp into our study (Yang et al. 2015, 2022) (supplementary fig. S2, Supplementary Material online), using GRAMPA may still lead to potentially incorrect autopolyploidization inferences, particularly in scenarios where both parental lineages of allopolyploids are extinct or inadequately represented in the sample. To address this issue, the genetic distance (*K*s) between ohnolog copies in each snow carp and their orthologs in the diploid species *Onychostoma macrolepis*, as well as in other well-documented allotetraploid Cyprinidae species, was calculated (Fig. 3c). Significant asymmetry in *Ks* values between ohnologs and their diploid orthologs, assessed in terms of subgenomes (Wilcoxon signed-rank tests, *P* ≤ 3.50 × 10^−33^), was consistently observed in allotetraploid species such as common carp, goldfish, and golden-line cavefish (panels in the first column, Fig. 3c). Conversely, in snow carp, ohnologs could not be definitively assigned to specific subgenomes, despite the availability of the snow carp genome (Xiao et al. 2020). Notable *K*s asymmetry between ohnologs relative to their diploid orthologs (Wilcoxon signed-rank tests, *P* ≤ 1.00 × 10^−3^) was only detectable when ohnologs were divided into two groups, with one group containing the ohnolog with a larger *K*s value in each pair and the other comprising those with smaller values. This pattern suggests that snow carp may be considered allopolyploids only under the highly unusual scenario where polyploidization occurred repeatedly within each deeply diversified matrilineal lineage, involving closely related diploids. Taken together, our results indicate that extant snow carp are primarily the result of recent and recurrent autopolyploidization events.

### Discussion

The most significant finding of our study is that extant polyploidy in snow carp species resulted from recent and recurrent autopolyploidization events, as opposed to one or two ancient polyploidization events. The number of independent autopolyploidization events identified in snow carp was notably and unexpectedly high. Our findings showed that independent autopolyploidization subsequent to speciation was common, while autopolyploidization preceding speciation also played a role in the diversification of snow carp. In our analysis, we identified 14 unambiguous autopolyploidization events among 18 of the more than 100 currently recognized snow carp species (Fig. 2e). This is likely an underrepresentation of the actual frequency of autopolyploidization in the diversification of snow carp, countering the long-held belief that one or two polyploidization events were fundamental in the origin of extant snow carp (Zan et al. 1985; Yang et al. 2015, 2022; Zhang, Tang et al. 2016; Li and Guo 2020). Furthermore, our results revealed that the instances of recurrent autopolyploidization in snow carp were surprisingly recent, occurring between 0.91 and 2.21 Mya during the Pleistocene (Fig. 2e; supplementary table S5, Supplementary Material online). This contrasts with earlier mitochondrial genomic studies that dated these events to between 36.90 and 15.00 Mya, spanning the Middle Miocene to Late Eocene (Li and Guo 2020; Yang et al. 2022; Gu et al. 2024). Notably, the recent and recurrent polyploidization events in snow carp remarkably contrasts with scenarios in other polyploid lineages in Cyprinidae, including common carp, goldfish, golden-line cavefish, and rock carp (*Procypris*), where a shared allopolyploidization event (about 14.00 to 12.00 Mya) is observed (Chen et al. 2019; Xu et al. 2019, 2023; Li and Guo 2020). To the best of our knowledge, this places snow carp as potentially the youngest lineage undergoing WGD (Ren et al. 2018). Additionally, our results indicate that recurrent autopolyploidization in snow carp is allopatric and widespread across the Tibetan Plateau and its surrounding areas (Fig. 2f), contradicting the theory that snow carp dispersed to their current habitats following the uplift of the Tibetan Plateau after polyploidization in common ancestors (Yang et al. 2015, 2022; Li and Guo 2020). Thus, the long-standing hypothesis that ancient polyploidization precedes the diversification of snow carp (Zan et al. 1985; Yang et al. 2015, 2022; Zhang, Tang et al. 2016; Li and Guo 2020) is refuted. Instead, our study suggests that recent autopolyploidization events in snow carp occurred independently post-diversification of their diploid progenitors which were already widely distributed on and around the Tibetan Plateau during its uplift.

The establishment of autopolyploidy in snow carp was a nonrandom process, closely associated with the extensive Pliocene–Pleistocene climate changes, including the Late Pliocene Transition (LPT, 3.00 to 2.50 Mya), the intensification of Northern Hemisphere glaciation (iNHG, 2.75 to 2.50 Mya), Pliocene–Pleistocene climate transitions (2.58 to 1.78 Mya), and the MPT (1.25 to 0.70 Mya). Generally, polyploids in nature are formed through the production of unreduced gametes, a process often stimulated by external factors, notably temperature fluctuations (Van de Peer et al. 2009; Rice et al. 2019; Fox et al. 2020; Novikova et al. 2020). The emergence of polyploidy in extant snow carp began after the iNHG, a period marked by a significant climatic shift from the warmth of the Late Pliocene to the global cooling of the LPT (Sosdian and Rosenthal 2009; Woodard et al. 2014). Notably, seven out of the 14 identified autopolyploidization events (Fig. 2e; supplementary table S5, Supplementary Material online) appear to have coincided with the MPT, a phase characterized by a shift in the Earth's glacial cycle periodicity from 41,000 to 100,000 years (Clark et al. 2006; Sosdian and Rosenthal 2009; Elderfield et al. 2012). During the Pliocene–Pleistocene climate transitions, global temperatures cooled gradually, albeit with significant fluctuations (Clark et al. 2006; Snyder 2016). On and around the Tibetan Plateau, diploid progenitors of extant snow carp likely faced even more pronounced temperature variations since the iNHG, potentially heightening the frequency of polyploidization occurrences in these species. Additionally, certain mating dynamics, such as dome matings, are recognized for producing more polyploids (Comai 2005). Furthermore, fossil evidence suggests that polyploid snow carp may have existed during the Oligocene, significantly predating the emergence of extant snow carp species (Yang et al. 2018; Yang, Liang et al. 2021). This implies that autopolyploidy may be an inherent strategy of their diploid progenitors in the face of global geological and climatic changes, since the process of autopolyploidy, involving genome doubling within a single species, is generally less complex than allopolyploidy which arises from the hybridization of two species followed by chromosome doubling (Comai 2005). In addition, autopolyploidy confers several key evolutionary advantages, such as increased gene dosage, enhanced genomic plasticity, and functional redundancy, which are critical in retaining beneficial mutations and evolving novel gene functions without compromising the integrity of existing ones, when adapts to rapidly changing environmental conditions (Comai 2005; Van de Peer et al. 2009; Rice et al. 2019; Fox et al. 2020). The benefits of polyploidy, notably an expanded genomic toolkit and asexual reproduction (Comai 2005), may have equipped polyploid snow carp to better adapt to harsh environments. This form of adaptability, as also seen in species such as *Neobatrachus* frogs (Novikova et al. 2020), has likely contributed to their enduring presence. Consequently, polyploidy appears to be uniquely observed in snow carp, as opposed to other fish species inhabiting the Tibetan Plateau and its environs (Yang, Wang et al. 2021), and the emergence of autopolyploidy in current snow carp may be the result of their diploid ancestors adapting to environmental and climatic fluctuations in and around the Tibetan Plateau during the extensive Pliocene–Pleistocene climate changes.

While our study elucidates a robust pattern of multiple independent autopolyploidization events in snow carp by integrating ohnolog genealogy, synchronization of speciation with polyploidization timelines, and historical biogeographical evidence, complex rediploidization processes after WGDs which have been frequently observed in fish (Robertson et al. 2017; Parey et al. 2022; Redmond et al. 2023) could theoretically lead to incorrectly distinguishing between shared and independent WGDs events even where the most of gene trees support independent WGDs events, particularly in ancient WGD lineages with slowly evolving genomes (Redmond et al. 2023), besides gene conversion whose effects are excluded in our study. This raises cautions for our understanding of the polyploidization history of closely related species based on ohnolog genealogy. Nevertheless, the recurrent autopolyploidization events in snow carp identified could be highly confident, given that shared polyploidization events are also identified in snow carp in our study with our data and methodologies. Additionally, slightly distinct timings of polyploidization events were observed across the three snow carp groups, correlating with specific climatic changes during the Pliocene–Pleistocene epoch, although ancestors of primitive, specialized, and highly specialized snow carp groups were facing the same Pliocene–Pleistocene climate changes. The primitive snow carp underwent polyploidization most recently (1.14 to 0.88 Mya), closely coinciding with the middle stage of the mid-Pleistocene Transition (MPT, 1.25 to 0.70 Mya), which suggests that they are the last to encounter climatic fluctuations. The specialized group, inhabiting mid-elevation ranges, experienced polyploidization slightly earlier (1.34 to 1.11 Mya) than primitive group during the earlier stage of the MPT. The highly specialized group, residing at the highest altitudes, underwent the earliest polyploidization events (2.34 to 1.21 Mya) which nearly encompasses the full Pliocene–Pleistocene climate transitions (2.58 to 1.78 Mya) and extends into the early MPT, suggesting that these species were the first to be impacted by the climate changes. These findings indicate that polyploidization events likely occurred independently across different groups, corresponding to their biogeographic distributions. As such, it would be interesting to know if primitive, specialized, and highly specialized groups are associated with independent or shared polyploidization in frequency. To do so, sampling to include most of snow carp species is asking and worth doing in the future. Finally, future high-quality genomes, e.g. subgenome-aware genome and haplotype-resolved assembly of autopolyploid genome whose assemblies are possible nowadays (Chen et al. 2024; Serra Mari et al. 2024; Sun et al. 2024; Wang et al. 2024), will not only validate findings of this study but also advance our understanding of the evolutionary dynamics of polyploidization and their adaptation to extreme environments in snow carp.

**Fig. 4.**
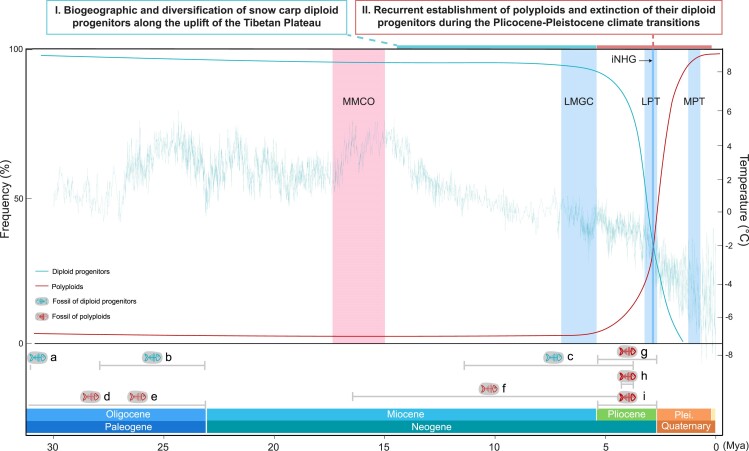
Establishment and diversification of extant snow carp with Tibetan Plateau uplift and paleoclimate changes. Long-term frequency dynamics of polyploids and their diploid progenitors represent a comprehensive scenario that integrates fossil records with our phylogenomic inferences. Global ice-free ocean temperature trends from the Oligocene to present are adapted from (Zachos et al. 2001). Known fossil records of polyploid snow carp and their diploid progenitors: a) *Tchunglinius tchangii* (Late Oligocene, 27.82 to 23.03 Mya) from Nima Basin (Wang and Wu 2015); b) Barbinae gen. et sp. indet. (Early Oligocene, 31.5 Mya) from Qaidam Basin (Chen and Liu 2007); c) *Plesioschizothorax macrocephalus* (Late Miocene or Early Pliocene, 11.63 to 3.60 Mya) from Lunpola Basin (Wu and Chen 1980; Chen 1998); d) *Paleoschizothorax qaidamensis* (Oligocene, 33.9 to 23.03 Mya) from Qaidam Basin (Yang et al. 2018); e) *Paleoschizothorax diluculum* (Oligocene, 33.9 to 23.03 Mya) from Qaidam Basin (Yang, Liang et al. 2021); f) *Schizopygopsis minutus* (Middle Miocene to Early Pliocene, 15.97 to 3.60 Mya) from Altai Mountains, Kazakhstan (Sytchevskaya 1989); g) *Hsianwenia wui* (Pliocene, 5.33 to 2.58 Mya) from Qaidam Basin (Chang et al. 2008); h) *Gymnocypris* sp., (Pliocene, 4.20 to 3.60 Mya) from Kunlun Pass Basin (Wang and Chang 2010); i) Schizothoracinae gen. et sp. indet. (Pliocene, 5.33 to 2.58 Mya) from Zanda Basin (Chang and Miao 2016). MMCO, Middle Miocene Climatic Optimum (17.00 to 15.00 Mya); LMGC, Late Miocene Global Cooling (7.00 to 5.40 Mya); iNHG, intensiﬁcation of Northern Hemisphere Glaciation (2.75 to 2.50 Mya); LPT, Late Pliocene Transition; MPT, Mid-Pleistocene Transition (1.25 to 0.70 Mya); Plei., Pleistocene.

This study presents a comprehensive scenario that illustrates the diversification of snow carp over time, incorporating matrilineal divergence, polyploidization events, fossil evidence, and paleoclimate changes (Fig. 4). While polyploids may have been present in this fish group since the Oligocene (Yang et al. 2018; Yang, Liang et al. 2021), their frequency was likely low. Environmental fluctuations may have increased the occurrence of polyploid snow carp, and the inherent advantages of polyploidy may have provided them with greater evolutionary fitness than their diploid ancestors during the extensive Pliocene–Pleistocene climate changes. Consequently, the extant snow carp emerged in the Pleistocene, long after their diploid progenitors had diverged from other Cyprinidae lineages in the Middle Miocene. Therefore, the diversification of current snow carp resembles a duet orchestrated by the uplift of the Tibetan Plateau and the extensive Pliocene–Pleistocene climate changes. The geographic changes brought about by the uplift of the Tibetan Plateau initially influenced the diversification of their diploid ancestors, followed by recurrent autopolyploidization during the Pliocene–Pleistocene climate changes, further driving the diversification of snow carp in the region surrounding the Tibetan Plateau. In summary, our study not only identifies snow carp as potentially the youngest WGD lineage, but also as a paradigm illustrating the adaptive, rather than random, nature of polyploid lineage establishment in evolutionary history not only in plants but also in vertebrates.

## Materials and Methods

### Sampling, Full-length Transcriptome Sequencing, and Genomic Data Assembling

Genomic data were collected from a total of 86 Cyprinidae species, including 67 snow carp (supplementary table S1, Supplementary Material online), covering currently known snow carp lineages and representatives of their most closely related diploid lineages (Yang et al. 2015, 2022). The genomic dataset included genomes from 10 Cyprinidae species, full-length transcriptomes from 25 Cyprinidae species, and 137 mitochondrial genomes from 78 Cyprinidae species. Publicly available genomic data were retrieved from GenBank, Genome Sequence Archive (GSA), and Ensembl. *De novo* full-length transcriptomes were determined for 14 snow carp species and seven diploid species using PacBio long-read isoform sequencing (Iso-Seq). The fish were sampled under ethical licenses in accordance with the national legislation in China and all experimental procedures approved by the Animal Care and Use Committee of the Institute of Zoology, Chinese Academy of Sciences (IOZ-IACUC-2020-115). To capture as many genes as possible, total mRNA from the brain, liver, and muscle of each species was pooled for Iso-Seq library construction for each species and sequenced using the PacBio Sequel Ⅱ platform with the multiplex sequencing strategy. The mRNA extraction, cDNA synthesis, Iso-Seq library construction, and sequencing were performed by Grandomics Biotechnology Co., Ltd (Wuhan, China). In total, ∼500 Gb of Iso-seq data were obtained from two SMRT cells, with 15 to 20 Gb Iso-seq data for each species. The raw Iso-Seq data were processed with SMRT Link v9.0.0 (Pacific Biosciences of California 2020). The number of high-quality nonredundant transcripts ranged from 91,823 to 210,706 across the 21 species, with three species having less than 100,000 transcripts. The significant difference in the number of transcripts among samples might be primarily due to variances of their RNA quality and the multiplex sequencing strategy with which multiple samples are sequenced in one SMRT cell and generated unequal sequencing data volume. The protein-coding region (CDS) for each species was determined using ANGEL v2.4 (https://github.com/PacificBiosciences/ANGEL). The completeness of transcriptome assemblies was assessed using benchmarking universal single-copy ortholog (Simão et al. 2015). Finally, 34,430 to 99,686 full-length transcripts with 5′- and 3′-untranslated regions and complete CDS were obtained for each species and used for subsequent analyses (supplementary table S1 and fig. S4, Supplementary Material online).

### Homolog Identification and Orthogroup Circumscription

Homolog identification was conducted using zebrafish (*Danio rerio*) genes as a reference with BLAST v2.2.26 (Altschul et al. 1997). Reciprocal BLAST analysis was run between zebrafish genes and transcripts from each species with an E-value of 1.00 × 10^−3^, no query sequence filtering (-F F), and no gapped alignment (-g F). BLAST hits with an E-value ≤ 10^−3^, coverage ≥ 50%, and identity ≥ 50% to a query sequence were grouped into a homolog family. Reciprocal BLAST analysis was then performed for each homolog family with the above-mentioned parameters to reduce transcript redundancy within each species. In cases where two transcripts within a species showed 100% identity in a homolog family, only the longest transcript was retained to represent a gene. Subsequently, the number of synonymous substitutions per synonymous site (*K*_S_) was calculated for genes represented by two or more transcripts within a species. If a gene in a species had two transcripts with a *Ks* value exceeding 1, the transcript that was the closest match to the zebrafish gene was preserved as the ortholog. However, if transcripts of a gene had a *K*_S_ value of 1 or lower, then these transcripts were retained as orthologs to the zebrafish gene. Finally, to minimize the impact of gene conversion which homogenizes genetic sequences, obscures their evolutionary origins, and distorts mutation rates, complicating the analysis of gene evolution and divergence (Chen et al. 2007; Mansai and Innan 2010), signals of gene conversion between ohnologs were identified using GENECONV v1.81a (Sawyer 1999) and ohnologs with signal of gene conversion were excluded in the following analyses in each snow carp species. The detailed workflow for homolog identification and orthogroup circumscription is provided in supplementary fig. S5, Supplementary Material online. A nearly identical *K*s distribution was observed between all 26,459 orthologous gene families and a subset of 7,202 orthologous gene families containing singleton genes in zebrafish (Inoue et al. 2015). This similarity was noted in paralog pairs of the snow carp *Schizothorax o’connori* (supplementary fig. S6, Supplementary Material online), aligning with the results of a previous study (Xiao et al. 2020) and affirming the accuracy of our ortholog identification process. Consequently, 26,459 orthologous gene families were established across 30 Cyprinidae species (supplementary fig. S7, Supplementary Material online).

### Ohnolog Genealogy Construction and Divergence Estimation

For each of the 26,459 orthologous gene families, multiple sequence alignment was conducted using MAFFT v7.310 (Katoh and Standley 2013), with ambiguous regions in the alignment removed using Gblocks v0.91b (Castresana 2000). Maximum-likelihood (ML) gene trees for each orthologous gene family were constructed using RAxML v8.2.12 (Stamatakis 2014), employing the -m PROT- GAMMAAUTO option for automatic selection of the best-fitting amino acid substitution model, 1,000 bootstraps for topology confidence assessment, and zebrafish genes as the outgroup. To determine whether snow carp originated from independent polyploidization events and/or shared polyploidization events, ML phylogenetic trees were generated for each orthologous gene family containing ohnologs from each pair of snow carp and four sets of snow carp triplets, respectively. To determine whether auto- and/or allopolyploidization occurred in snow carp, an algorithm for topology-based gene-tree reconciliation was applied, utilizing multilabeled trees tailored for polyploid analysis in GRAMPA. A species tree for snow carp and their closely related diploid relatives (supplementary fig. S2, Supplementary Material online) was inferred using a coalescent-based approach implemented in ASTRAL-Pro (Zhang et al. 2020), using ML gene trees derived from orthologous gene families to ensure a dataset with fewer than 10 missing taxa. In GRAMPA analysis, gene trees containing the focal snow carp and their most closely related diploid relatives were reconstructed, and the species tree generated by ASTRAL-Pro was pruned to only include focal species. The gene tree sets were visualized using DensiTree v2.2.7 (Bouckaert and Heled 2014). For each pruned species tree, all possible multilabeled trees were scored, with a maximum of 15 polyploid groups allowed. The reconciliation scores of the optimal multilabeled trees were then compared to those of the single-labeled species tree. Notung v2.9.1.5 (Durand et al. 2006) was used for ﬁnding the most parsimonious topologies around nodes with low bootstrap support and for conducting bootstrap rearrangement in GRAMPA analysis with triplets of snow carp. Finally, the *K*_S_ values between ohnologs in each orthologous gene family for each species were calculated using codeml in PAML v4.9 h (Yang 2007). A synonymous substitution rate (*r*) of 3.51 × 10^−9^ substitutions per synonymous site per year was used as the mutation rate in Cyprinidae (Graur and Li 1997). Divergence time (*T*) between ohnologs in each snow carp species in each orthologous gene family was calculated using the equation *T* = *K*_S_/2*r*.

### Mitochondrial Phylogeny Inference and Divergence Time Estimation

A concatenated alignment of 13 mitochondrial protein-coding genes from 137 mitochondrial genomes representing 78 Cyprinidae species (supplementary table S1, Supplementary Material online) was performed using MAFFT. The mitochondrial phylogeny of snow carp was inferred using the ML method, with the best-fit model automatically selected by ModelFinder (Kalyaanamoorthy et al. 2017) and ultrafast bootstrapping (-bb 1 000) in IQ-TREE v2.0.3 (Minh et al. 2020), using zebrafish as the outgroup. Divergence times among snow carp were estimated using MCMCTree in PAML based on the above-mentioned ML tree. Time calibration incorporated three constraints: (i) a minimum age constraint of 16.00 Mya was assigned to the most recent common ancestor (MRCA) of Barbinae and Labeoninae (node of C1, Fig. 1), following the earliest fossil records of *Labeo*-like and *Barbus*-like fish in the Early Miocene (Van Couvering 1977; Stewart 2001); (ii) a minimum age constraint of 5.33 Mya was assigned to the root of Labeoninae (node of C2, Fig. 1), corresponding to the fossil record of *Labeo* species in the Late Miocene (Van Couvering 1977; Stewart 2009); (iii) a maximum age constraint of 0.15 Mya was assigned to the MRCA of two snow carp, *Gymnocypris przewalskii* and *Gymnocypris eckloni* (node of C3, Fig. 1), reflecting the separation of Qinghai Lake and the Yellow River around 0.15 Mya (Zhang et al. 2003). The MCMC analysis included an initial burn-in of 2,000 iterations, followed by 20,000 additional iterations with a sampling frequency of 10. This process was repeated twice to check for consistency between runs. The resulting time-calibrated tree was visualized using FigTree v1.4.4 (http://tree.bio.ed.ac.uk/software/figtree).

## Supplementary Material

msae221_Supplementary_Data

## Data Availability

The raw Iso-Seq data (GSA: CRA012856) determined in this study have been deposited in the GSA in National Genomics Data Center, China National Center for Bioinformation/Beijing Institute of Genomics, Chinese Academy of Sciences, and are publicly accessible at https://ngdc.cncb.ac.cn/gsa. All data used in the study are provided in supplementary table S1, Supplementary Material online.
