# Supplementary material for: Recent and Recurrent Autopolyploidization Fueled Diversification of Snow Carp on the Tibetan Plateau
Source: Mol Biol Evol. 2024 Oct 22;41(11):msae221. doi: 10.1093/molbev/msae221 (PMC11542630; doi:10.1093/molbev/msae221)
Supplement: msae221_Supplementary_Data [file msae221_supplementary_data.zip › SupplementalData-Figures.pdf]

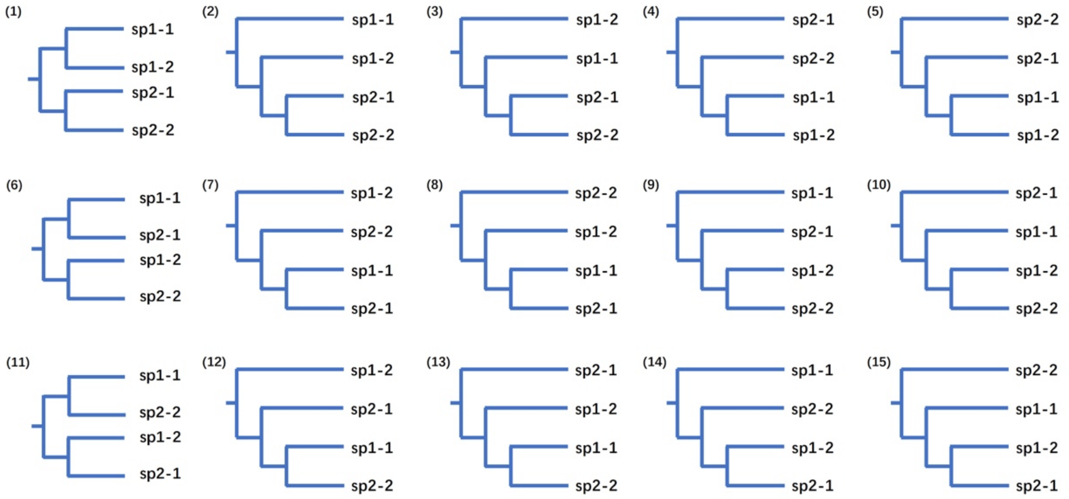

Figure S1 The 15 rooted topologies of a four-tips tree with sp1-1 and sp1-2 from species 1#, and sp2-1 and sp2-2 from species 2# in an ohnologous gene family.

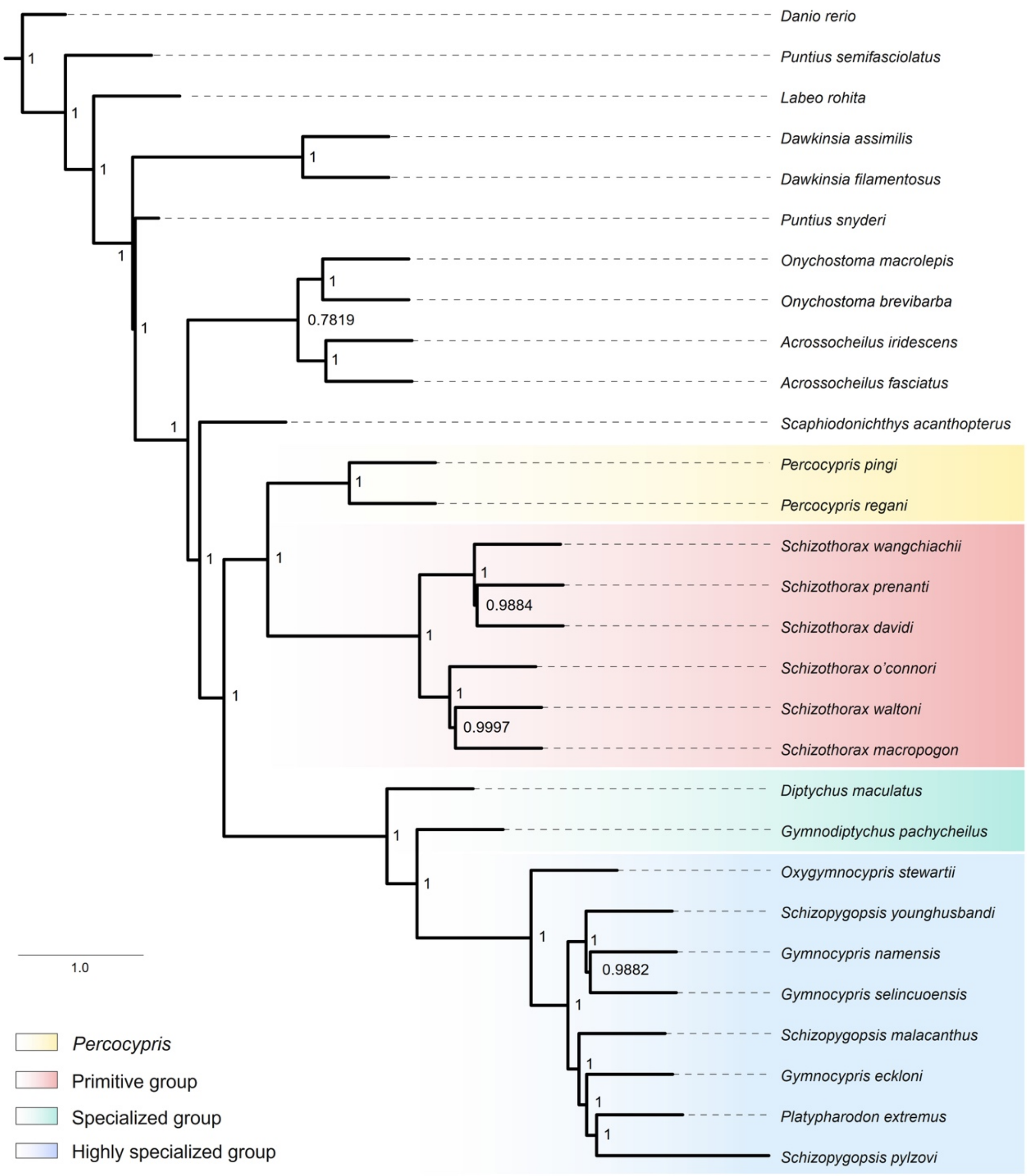

Figure S2 The inferred Astral-Pro tree. Local posterior probability (LLP) is shown next to the nodes. Branch lengths are in coalescent units.

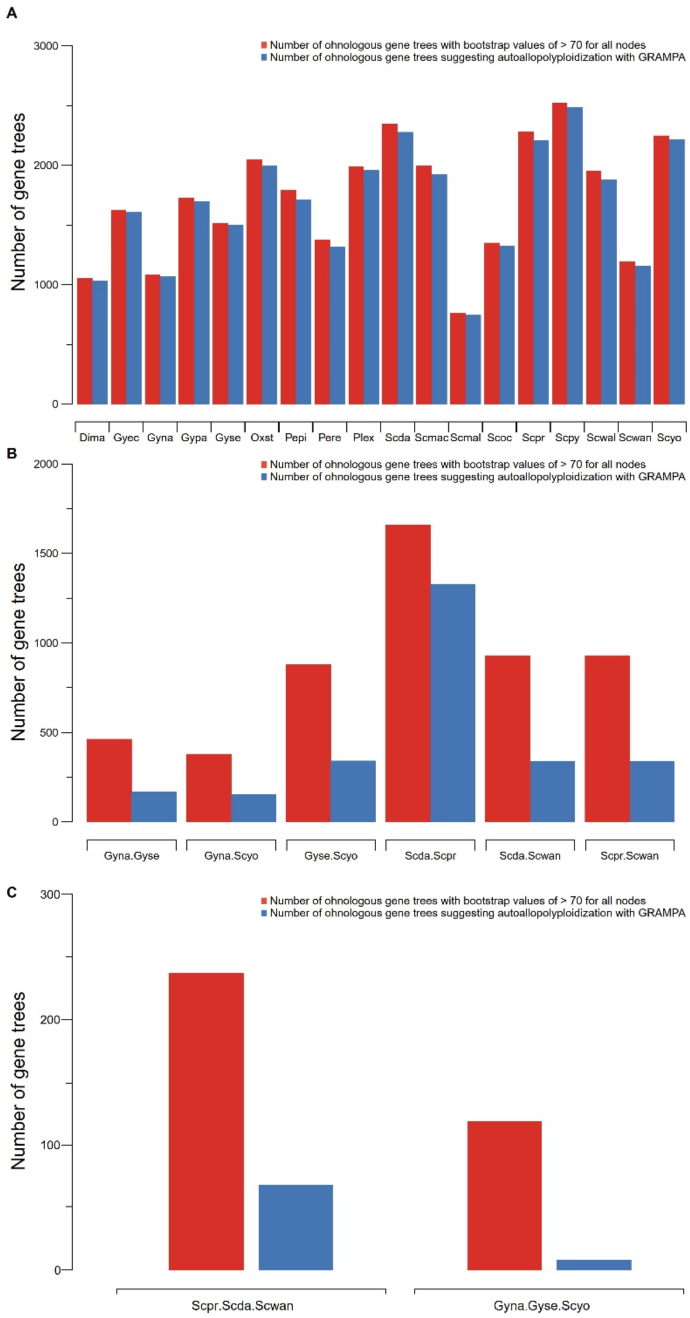

Figure S3 Number of MUL-trees used in GRAMPA.

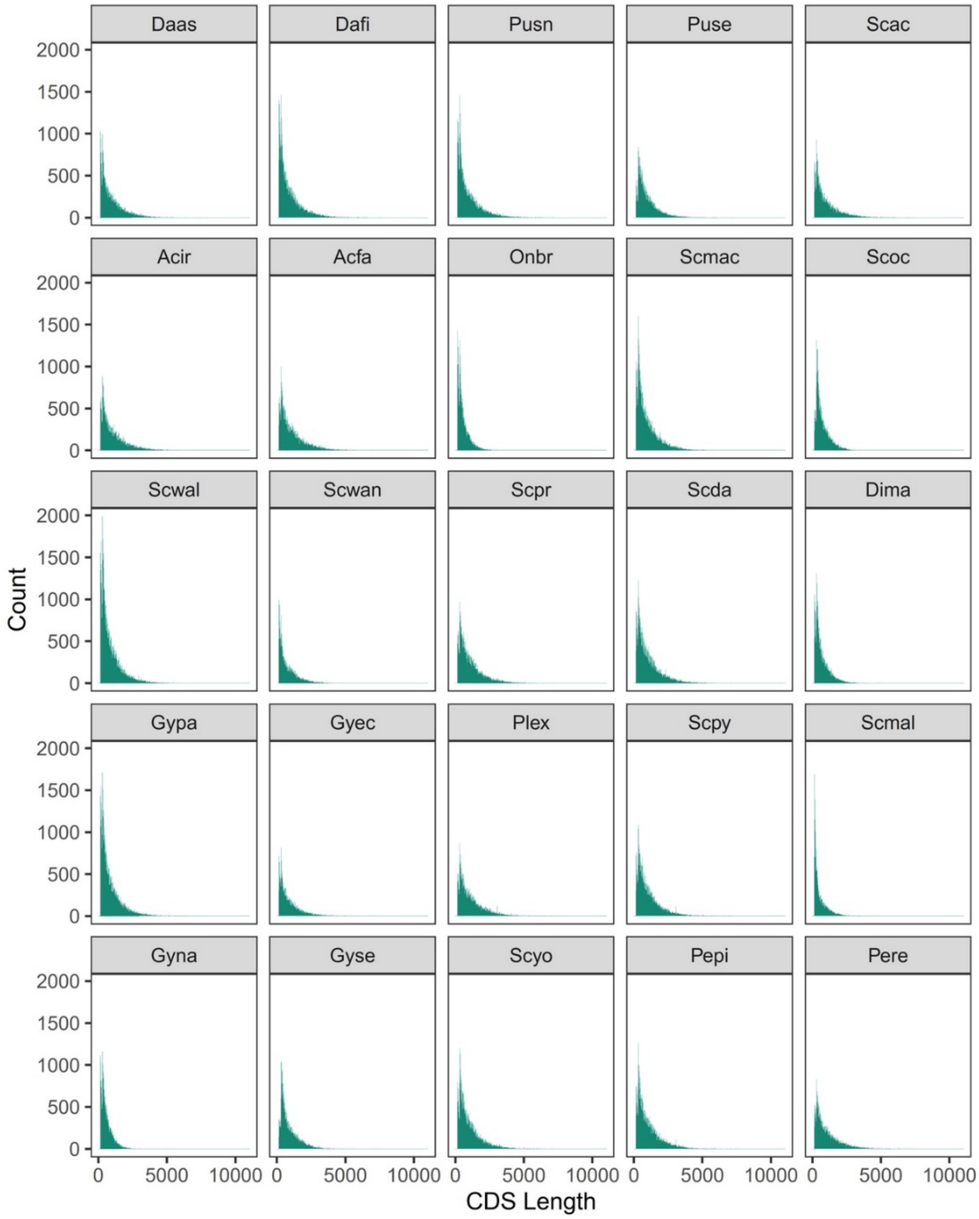

Figure S4 The CDS length distribution in snow carps and their diploid relatives.

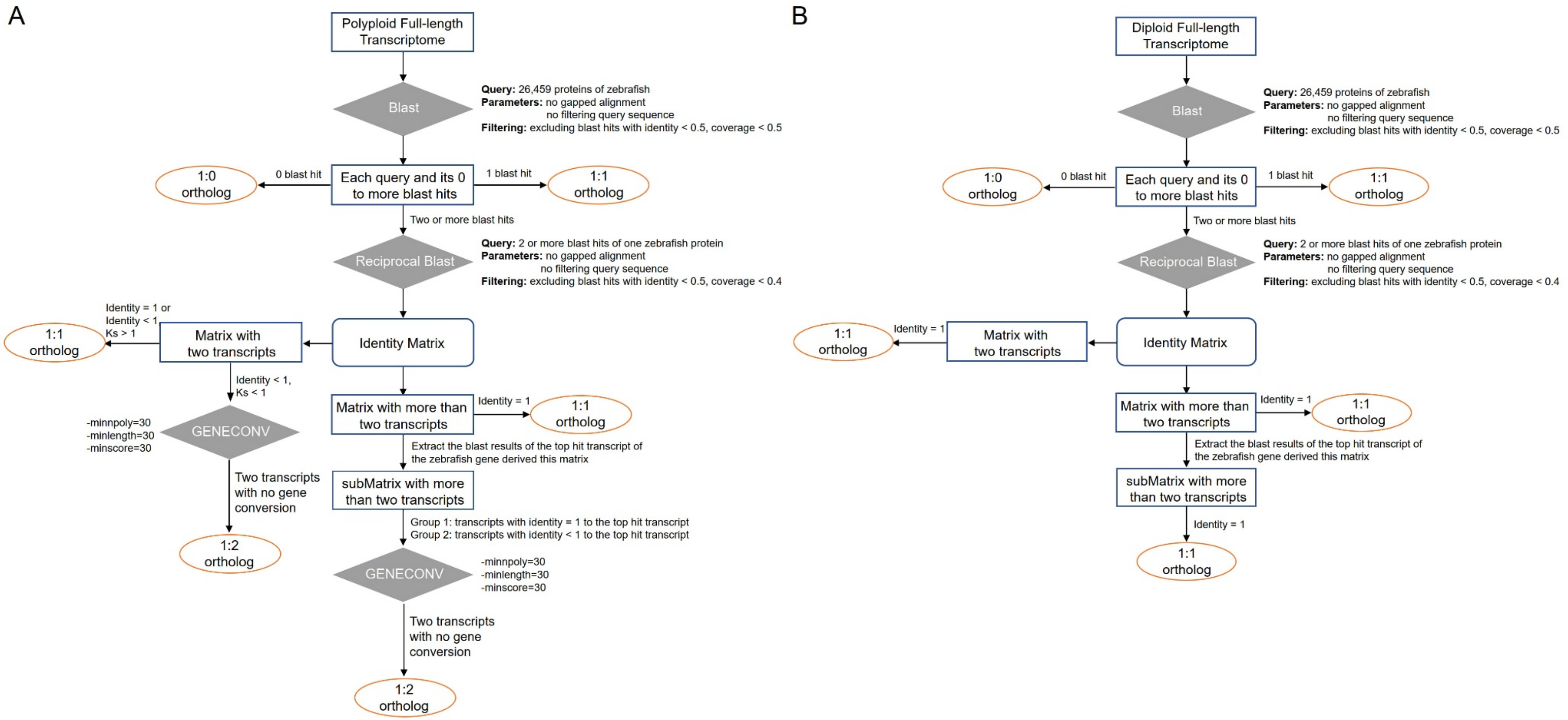

Figure S5 The pipeline used for homolog identification and orthogroup circumscription.

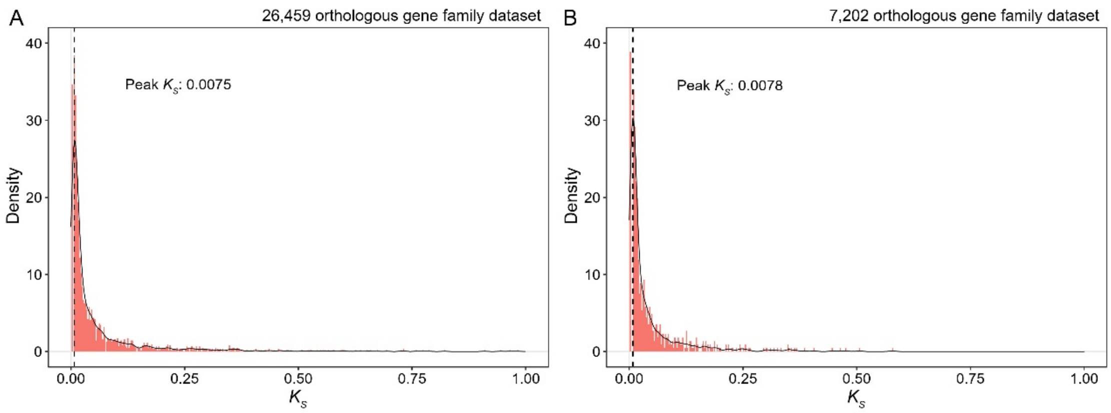

Figure S6 The K_S distribution and the peak K_S value for homologous pairs in *Schizothorax o'connori* based on all (26,459) orthologous gene families and 7,202 orthologous gene families with single copy gene in zebrafish.

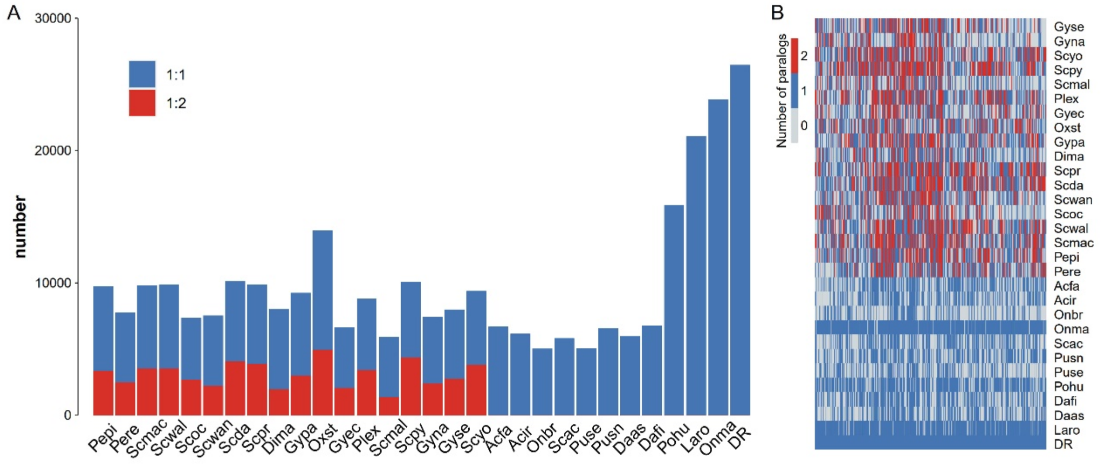

Figure S7 The characteristics of the inferred orthogroups across 18 snow carps and 13 diploid species. (A) The number of 1to1 (red) and 1to2 (blue) orthogroups in each species. (B) The number of paralogs per taxa in the inferred orthogroups. Grey, blue, and red indicate zero, one, and two paralogs, respectively.
